# Monoclonal Gammopathy of Renal Significance and Thrombotic Microangiopathy: A Case Report

**DOI:** 10.7759/cureus.32753

**Published:** 2022-12-20

**Authors:** Sofia Ventura, Raquel Cabral, Carolina Viveiros, Mariana S. Santos, João Esteves

**Affiliations:** 1 Nephrology, Hospital do Divino Espírito Santo, EPER, Ponta Delgada, PRT; 2 Hematology, Hospital do Divino Espírito Santo, EPER, Ponta Delgada, PRT; 3 Intensive Care Unit, Hospital do Divino Espírito Santo, EPER, Ponta Delgada, PRT

**Keywords:** retroperitoneal hematoma, kidney biopsy, subnephrotic proteinuria, haemolytic anaemia, rapidly progressive renal failure, dialysis, plasma therapy, monoclonal gammopathy of renal significance, thrombotic microangiopathy (tma)

## Abstract

Monoclonal gammopathy of renal significance (MGRS) is a group of pathologies that includes all kidney disorders related to a monoclonal protein in patients without diagnostic criteria for B-cell malignancies. There are multiple MGRS-associated kidney disorders, and more are still being discovered, which makes this diagnosis challenging. The relationship between monoclonal gammopathies and thrombotic microangiopathy (TMA) is of growing interest in literature. This article describes the case of a patient with newly diagnosed MGRS, presenting with rapidly progressing kidney failure and with histologic characteristics of TMA. The patient progressed to end-stage renal disease (ESRD) despite treatment with plasmapheresis and clone-directed therapy, as is currently advised in the literature. Although rare, the association between these two entities should not be unnoticed because of patients’ renal and vital prognosis.

## Introduction

During the assessment of patients presenting with kidney disease, it is standard procedure to screen for monoclonal proteins, with the first-line diagnostic tools being the investigation of serum free light chains and the performance of serum protein electrophoresis with immunofixation [1]. When a monoclonal protein is present in patients who don’t fit the criteria for multiple myeloma or lymphoproliferative disorders, it is necessary to distinguish between monoclonal gammopathy of undetermined significance (MGUS) and monoclonal gammopathy of renal significance (MGRS) [2-3].

MGUS is an asymptomatic premalignant condition characterized by the presence of an immunoglobulin from a clone of bone marrow plasma cells without attributable organ damage [1,4]. This monoclonal protein, denominated the M-protein or paraprotein, by definition, is required to have a concentration inferior to 3g/dL, with patients presenting less than 10% bone marrow plasma cells [1,4]. Since this is a benign condition, patients with MGUS do not meet any current hematologic criteria for specific therapy [2-3].

Differently, MGRS is an entity recognized by the International Kidney and Monoclonal Gammopathy Research Group (IKMG) since 2012, encompassing a group of pathologies in which the M-protein is related to the pathogenesis of kidney disease [2-4]. This condition’s diagnosis is often challenging because of its broad spectrum and the difficulty in establishing a connection between the organ damage and the presence of the M-protein or serum free light chains [2]; in fact, this causal relationship is not related to the hematological criteria and is usually evaluated through renal histology [1,4]. Renal disease in MGRS may emerge as the first clinical statement of a monoclonal gammopathy or may appear as a complication of a previously diagnosed hematologic disorder (including MGUS or smoldering myeloma) [1]. These patients may have acute, subacute, or chronic kidney diseases, electrolyte abnormalities, proteinuria, and/or nephrotic syndrome [1]. Most commonly, they present with kidney impairment and proteinuria, with or without hematuria [1].

Despite its nonmalignant nature, MGRS has significant morbidity and mortality [2]. Even though mortality is lower than in patients with multiple myeloma, the renal outcomes are worse, and the patients with MGRS are at increased risk of progression to end-stage renal disease (ESRD) [2,4]. Therefore, treatment targeting the B-cell or plasma cell clone that produces the M-protein should be provided, aiming to preserve organ function and prevent further progression of damage [1-3].

Various kidney diseases are recognized as being associated with monoclonal gammopathy, either single or in combination, and this list is still increasing [2]. Most MGRS cases are due to direct deposition of the immunoglobulin fragments or whole immunoglobulins with different locations and patterns of ultrastructural organization, resulting in well-characterized glomerular injuries either with organized deposits (fibrillar or microtubular) or nonorganized deposits - including AL amyloidosis, monoclonal immunoglobulin deposition disease, and proliferative glomerulonephritis with monoclonal immunoglobulin deposits [2,4-5]. Indirect mechanisms by which monoclonal gammopathies cause kidney injury include dysregulation of the alternate pathway of complement, which may result in C3 glomerulopathy or, rarely, thrombotic microangiopathy (TMA) [5]. Other vascular patterns may also be found, as well as tubulointerstitial disease (including, for example, Fanconi syndrome) [2,4].

The authors report the case of a patient with histologically documented TMA associated with MGRS. TMA comprises a heterogeneous group of diseases characterized by microvascular cell injury caused by platelet-rich and/or fibrin thrombi occluding small vessels of various organs, mainly the kidney and the brain [5]. Systemic features, such as microangiopathic hemolytic anemia (MAHA) and thrombocytopenia, may be present; nonetheless, the disease may be kidney limited, with the renal biopsy playing a pivotal role in those cases [5]. The TMA may be primary (congenital or acquired), namely thrombotic thrombocytopenic purpura (TTP) or atypical hemolytic uremic syndrome (aHUS), or secondary to numerous drugs or diseases, including infections (particularly Shiga-toxin producing *Escherichia coli*, STEC), autoimmune conditions, metastatic cancer, malignant hypertension, pregnancy, and transplantation [5]. Different secondary causes can coexist and may also act as triggers for individuals with underlying genetic susceptibility to a primary TMA [5].

## Case presentation

This was a 68-year-old Caucasian woman, with a personal history of dyslipidemia, osteoporosis, and bilateral carpal tunnel syndrome. She presented in the Rheumatology clinic with asthenia and *de novo* arterial hypertension. She was started with an angiotensin-converting enzyme (ACE) inhibitor and calcium channel blocker and proceeded the investigation with an ambulatory blood pressure measurement and an ocular angiography, which revealed, respectively, median blood pressure values of 143/100mmHg and hypertensive retinopathy. Blood analysis revealed mild anemia (hemoglobin [Hb] 11.4g/dL), with normal kidney function (creatinine 0.64mg/dL), protein electrophoresis, thyroid function, and broad autoimmune markers, as well as a normal urinalysis and urinary catecholamines assessment (Table 1). Six months later, the analytic evaluation showed worsening of the anemia (Hb 8.2g/dL) and of the kidney function (creatinine 2.99mg/dL); urinalysis presented with proteinuria and hemoglobinuria, and urinary sediment showed microhematuria (5 red blood cells per high power field) without leukocyturia. The platelet count was normal. Urinary creatinine clearance (24-hour urine sample) was 17mL/min/1.73m^2^. One month later, she presented with Hb 7.8g/dL, creatinine 4.71mg/dL, and urinary creatinine clearance of 11ml/min/1.73m^2^, consistent with rapidly progressive kidney failure. At this moment, she was referred to Nephrology and admitted to the hospital for a complementary study.

**Table 1:** Evolution of the patient's analytical evaluation since the arterial hypertension diagnosis. Hb: hemoglobin

First medical evaluation	6 months later	7 months later
Hb 11.4g/dL. Normal leukocyte and platelet counts	Hb 8.2g/dL. Mild leukopenia, no thrombocytopenia	Hb 7.8g/dL. No leucopenia nor thrombocytopenia
Urea 32mg/dL; Creatinine 0.64mg/dL	Urea 142mg/dL; Creatinine 2.99mg/dL	Urea 137mg/dL; Creatinine 4.71mg/dL
Normal urinalysis	Urinalysis with proteins (+) and hemoglobin (+); urinary sediment with microhematuria, without leukocyturia	Non-nephrotic proteinuria: 1.5g/24h; urinary sediment with microhematuria, without leukocyturia
Protein electrophoresis, urinary catecholamines, thyroid function - normal	Sedimentation rate 48mm Urinary creatinine clearance (24h) – 17mL/min/1.73m^2^	Urinary creatinine clearance (24h) – 11mL/min/1.73m^2^

In the inpatient clinic, we detected non-nephrotic proteinuria (1.5g/day), as well as the presence of polyclonal cryoglobulins (IgA, IgM, and IgG) and elevated serum free light chains (Kappa and Lambda), with monoclonal IgG/Kappa spike in serum immunofixation (2.5% - 0.1g/dL). There were no schistocytes in the blood smear; platelet count was normal, as well as lactate dehydrogenase (LDH) and serum calcium levels. Viral serologies, including human immunodeficiency virus (HIV), hepatitis C virus (HCV), and hepatitis B virus (HBV), were negative; autoantibodies (antineutrophil cytoplasmic antibodies [ANCAs], antinuclear antibodies [ANAs], anti-double-stranded DNA antibodies [anti-dsDNA], anti-glomerular basement membrane [anti-GBM] and antiphospholipid antibodies), complement levels, and immunoglobulin levels were normal. For the complementary study of the monoclonal spike, we performed a skeleton radiography, showing the absence of lytic lesions. Both a myelogram and a bone marrow biopsy were performed, revealing a normal myeloid to erythroid ratio (2.6:1) and a low percentage of plasma cells (1.8%). We assumed the initial diagnosis of cryoglobulinemia associated with MGRS.

The patient's renal function continued to decline, and hemodialysis (HD) was initiated on the 13^th^ day of internment, via a jugular central venous catheter (CVC). No anticoagulation was used in the circuit during HD sessions, and the catheter branches were filled with citrate. A renal biopsy was performed on the 14^th^ day of internment, ultrasound-guided, without immediate complications.

On the 16^th^ day of internment, the patient developed hemolytic anemia, with thrombocytopenia, elevated LDH, low haptoglobin, and the presence of schistocytes and reticulocytes in blood smear. As direct Coomb’s test was positive, an episode of autoimmune hemolytic anemia was assumed.

**Figure 1:**
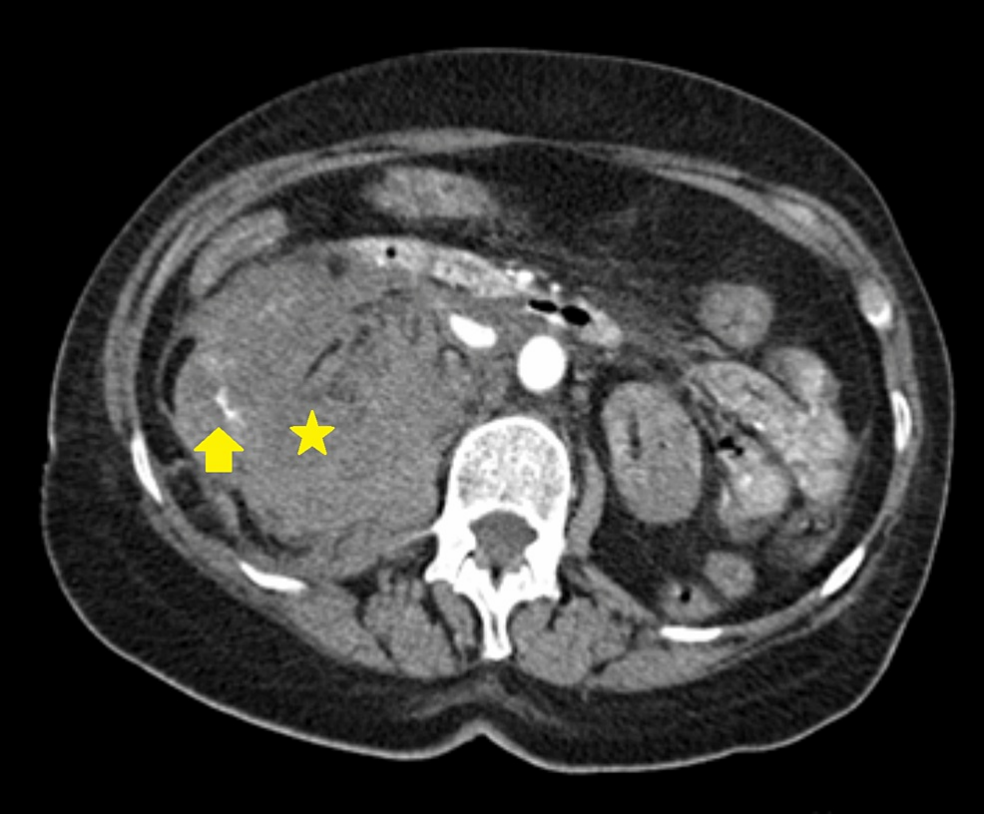
Right-sided renal and retroperitoneal hematoma - CT scan, transversal cut The hematoma (yellow star) occurred as a late complication of the kidney biopsy - four days after the procedure. The yellow arrow points toward the active extravasation of material, which suggests active hemorrhage.

**Figure 2:**
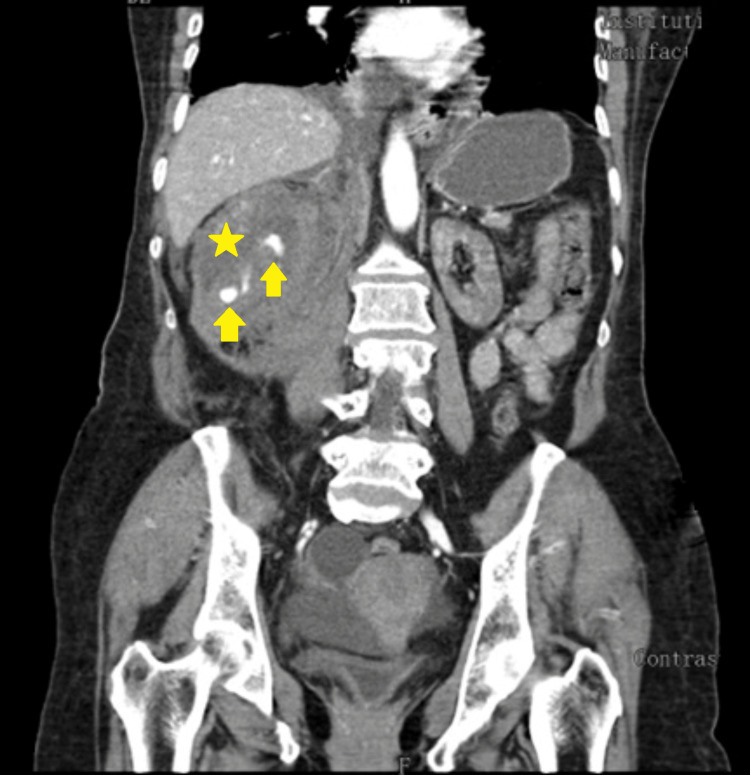
Right-sided renal and retroperitoneal hematoma - CT scan, coronal cut The hematoma (yellow star) occurred as a late complication of the kidney biopsy - four days after the procedure. The yellow arrows point toward the active extravasation of material, suggesting active hemorrhage.

A clinical picture of hemorrhagic shock developed on the 18^th^ day, with radiological evidence (CT scan) of renal and retroperitoneal hematoma emerging from the inferior pole of the kidney (superficial orifice with about 2mm) - we assumed a later complication of the renal biopsy (Figures 1-2). The case was discussed with Urology and Vascular Surgery and was considered only for medical treatment. The patient was transferred to the Intensive Care Unit (ICU), received blood transfusions (frozen plasma and erythrocyte concentrates), and was started on hydrocortisone in shock-dosing.

The renal histology showed evidence of TMA, and the patient was started on plasmapheresis (initiated on the 22^nd ^day) - it was then considered the diagnosis of TMA associated with MGRS and, with the collaboration of Hematology, therapeutic with bortezomib, cyclophosphamide and dexamethasone was instituted. Both a genetic study of the complement cascade and ADAMTS13 activity levels were obtained before plasmapheresis initiation and showed normal results. Regarding other TMA etiologies, we highlight the negative autoimmune testing (as previously described), the absence of clinical or analytical signs of infection, as well as negative viral serologies and blood and urine cultures, the presence of reasonable blood pressure control while in the hospital, and the absence of use of drugs usually associated with this condition.

Because there was no evidence of renal recovery, chronic HD treatment was maintained. Without other relevant occurrences, hospital discharge happened after 40 days, following three complete cycles of chemotherapy. In the ambulatory clinic, the patient maintained chemotherapy and a regular HD program, assuming end-organ damage (ESRD). In the two months following discharge, important infectious complications occurred, namely bacterial pneumonia and CVC infection; parallelly, she maintained no signs of renal function recovery. Thus, the analysis of the risk-benefit profile prompted the discontinuation of chemotherapy, proceeding only with dialytic support.

## Discussion

We describe a complex case of thrombotic microangiopathy and monoclonal gammopathy in which we highlight the importance of the renal biopsy since the clinical picture was unrevealing of the putative diagnosis, as the patient did not present with systemic features of TMA. One of the factors that made the diagnosis even more difficult was the occurrence of autoimmune hemolytic anemia and hemorrhagic shock.

Concerning the TMA occurrence, in this case, we will now discuss in extensive detail the evaluation of the possible etiologies and triggers. The most plausible hypothesis in our patient, as previously described, is the association with MGRS, keeping in mind the compatible clinical picture of microhematuria, proteinuria, and rapidly progressive kidney function; the progression to ERSD and non-reversibility despite the treatment aligns with the poor renal prognosis of these situations [2,4,6]. With concerns to aHUS, our patient’s complement pathway study did not show the presence of mutations; however, it is not possible to completely rule this option out because, as it is known, about 30-40% of patients with aHUS have no identified mutations [7]. TTP appears as a less likely possibility, as the patient had normal ADAMTS13 activity levels; however, we were not able to test for antibodies against this enzyme, which could also be of interest. The autoimmune disease appears as one of the possible diagnoses of this patient, particularly because of the occurrence of autoimmune hemolytic anemia on the 16^th^ day of internment in a patient that had previously been diagnosed with bilateral carpal tunnel syndrome; however, the authors would like to underline the negative result of the broad autoimmune panel that was performed, including complement levels, ANCAs, ANAs, anti-dsDNA, anti-GBM, and antiphospholipid antibodies. Infectious causes of TMA were also discarded since the patient had no clinical or analytical signs of infection and had negative viral serologies. Another ruled-out hypothesis was hypertension-associated TMA, as the clinical picture did not resolve with blood pressure control, as would be expected [8]. The same goes for drug-associated TMA, as the patient wasn’t under treatment with suspect drugs (such as calcineurin inhibitors, interferon, anti-aggregating agents, or quinine [9]), nor in ambulatory nor after in-hospital admission.

The relationship between TMA and monoclonal gammopathies is of growing interest [6,10], and TMA was attributed by the IKMG a provisional status as one of the kidney lesions associated with MGRS [11]. Despite being a rare complication, monoclonal gammopathy has an unexpectedly high prevalence in patients with TMA, suggesting a potential association between these two entities - in the largest case series, Ravindran et al. retrospectively analyzed 146 patients with MAHA or biopsy-proven TMA that had been screened for paraproteins and found that 20 patients (13.7%) were positive, making the incidence of paraproteinemia with TMA four times higher than in the general population [6]. In this sub-population [6], the clinical presentation included hematuria, proteinuria, and a decline in renal function, similar to what we observed in our patient. In a smaller case series [10] including nine patients with paraprotein-associated TMA, the median age was 66 years old, and three (33.3%) were female; all patients had renal involvement.

The mechanisms by which monoclonal gammopathy-associated TMA occurs are still under investigation. It is thought that the paraproteins may either work as triggers in individuals with underlying susceptibility, be the sole cause of the process, or act as underlying susceptibility factors [5]. It is hypothesized that the development of TMA may be facilitated either directly or indirectly by the paraprotein, as it is known that its direct deposition in the kidney does not occur [5]. Suggested direct mechanisms include direct injury to the endothelial cells or interference with the fibrin structure, for example, through interactions between the paraprotein and platelet membrane glycoprotein 1b or von Willebrand factor [6,10]. Indirect mechanisms involve functional inhibition of thrombosis-regulating proteins and/or dysregulation of the alternate pathway of complement [5-6,10], as was previously stated.

In MGRS-associated TMA, the first-line treatment must encompass disease-directed therapy [2-5]. Although studies on the clone-directed treatment of MGRS did not show a life-expectancy increase, hematologic remission may induce a kidney remission that delays or prevents progression to ESRD [5]. However, we highlight that in one of the aforementioned studies [6], despite treatment implementation, ESRD developed in 50% of the cases (10 patients), suggesting that this subpopulation may have a poorer renal outcome than patients with TMA without monoclonal gammopathy.

Another therapeutic approach that could be beneficial in paraprotein-related TMA is pharmacological C5 inhibition, keeping in mind that complement system dysregulation is one of the putative pathogenic mechanisms for the disease [5]. However, the role of this therapeutic is controversial in literature - if the observed dysregulation is, in fact, paraprotein-mediated, the induction of hematologic remission (by clone-specific therapy) should probably be sufficient for treatment purposes [5]. Thus, it is advocated that C5-inhibitors' usage should only be considered in the presence of severe, life-threatening disease involving other organs [5]. We decided not to perform this treatment on our patient since she had already progressed to ESRD and showed no evidence of recovery with the implemented measures.

## Conclusions

Although a rare diagnosis, monoclonal gammopathy-associated TMA should not be overlooked, with description cases playing an important role in highlighting the possible clinical presentations, treatment schemes, and outcomes. When in presence of a renal-limited TMA, paraprotein disorders should not be overlooked, even though additional studies would be necessary to determine in further detail the mechanisms between these two entities. In this group of patients, the treatment of TMA should always include targeting the monoclonal gammopathy, with implications for patients’ renal and vital prognosis; C5 inhibition may also be considered in specific cases, although controversial.
